# Exploring research and healthcare priorities in maternal health: A qualitative ethnographic study with mothers from ethnic minority backgrounds in the UK

**DOI:** 10.18332/ejm/209195

**Published:** 2025-09-12

**Authors:** Amy Furness, Alison Salmon, Frankie Fair, Hora Soltani

**Affiliations:** 1 College of Health, Wellbeing and Life Sciences, Sheffield Hallam University, Sheffield, United Kingdom

**Keywords:** pregnancy, postnatal, ethnicity, socioeconomic inequality, maternal health disparities, cultural barriers

## Abstract

**INTRODUCTION:**

Despite national efforts, inequalities in maternal and infant health persist. Black, Asian and other ethnic minority, along with those in deprived areas, face disproportionately high complication and mortality rates. Prioritizing research is crucial for improving care experiences for women and families.

**METHODS:**

By adopting a qualitative ethnographic approach, we explored priority areas for research regarding Black, Asian, and ethnic minority mothers accessing healthcare in the UK. Data were gathered through focus groups and analyzed inductively and thematically using NVivo. The study sample comprised 55 women from various ethnic backgrounds, with the largest groups identifying as Black African, Arab, and Asian Pakistani.

**RESULTS:**

Women outlined key research priorities stemming from significant challenges in accessing maternity care. These included: 1) Communication barriers such as language difficulties, understanding each other and health literacy; 2) Emotional and psychological support, highlighting a need for further research. Women underscored the value of safe spaces for peer support and social interaction; 3) Participants stressed the importance of comprehensive perinatal education, particularly during the transition to parenthood, along with a strong desire for digital resources, information sharing, and networking; 4) High-quality, compassionate, and well-coordinated maternity care remained a primary concern; and 5) Socioeconomic support, including financial assistance, childcare, and resources for essential postnatal needs.

**CONCLUSIONS:**

To tackle these issues, research at both community and individual levels should be commissioned to ensure women’s priority concerns are comprehensively addressed. These findings provide valuable insights that can help shape national efforts to improve maternity care and reduce disparities, by informing policy and improving professional training.

## INTRODUCTION

Despite national policy initiatives, outcome disparities persist for Black and Asian women, especially those in socially deprived areas^1,2^. Data highlight key factors driving maternal and infant inequalities, including mental health, obesity, and higher stillbirth and neonatal mortality rates, disproportionately affecting Black and Asian infants^1,3^.

Recent evidence suggests a range of underlying causes of these health inequalities. Reports indicate concerns about the accessibility of healthcare systems and include a narrative of not feeling listened to; a lack of information and instances of stereotyping^3,4^. Maternal inequalities stem from structural and interpersonal racism, compounded by social and economic disadvantage^5^.

There is a large body of evidence suggesting midwives experience challenges whilst caring for ethnic minority women^6^. Notably, these women experience communication and translation issues which restrict their access to services^6-8^. Health literacy among pregnant women is low, with lower levels observed among Black, Asian, and ethnic minority groups and those from lower socioeconomic backgrounds^9,10^. Little has been done to improve health literacy within this demographic, leading women to seek information from friends, family, and online sources, which perpetuates inequalities in maternal and infant care^10^.

To address significant inequities, it is essential to understand the needs and priorities of ethnic minority women. These needs extend beyond maternity care to include social and physical well-being^8^. There has been growing recognition of the importance of perinatal mental health among ethnic minority women. Research indicates that pregnant women who have recently immigrated to the UK often experience poor social support networks, increasing their risk of isolation^8,10,11^, highlighting the need for effective interventions developed in consultation with women to ensure they meet their needs^11^.

Previous research has established the importance of kind and compassionate culturally safe care^3,4^. In addition, continuity of care and women-centered care is known to reduce health inequalities, particularly for disadvantaged groups^3,4^. A growing body of literature recognizes the need to improve training and education for professionals in diagnosing conditions and deterioration based on skin color and contribute to practical and policy changes being applied to decolonize midwifery^4,12^. However, there is a paucity of research exploring the evidence for successful interventions at the community level and attempts to transform organizational culture have not made meaningful change^5^.

A multi-interventional approach is needed that recipients will find acceptable, culturally relevant, sensitive and respectful^13^. Yet, without the appropriate evidence base such community interventions and corresponding improvement in health outcomes may be limited^7^. Research dictates that women living in the most deprived areas and those from Black, Asian and other ethnic minority groups are less likely to be included in research^14^. Addressing biomedical factors alone is insufficient, research into the needs and care of minoritized ethnic perinatal women, at a local level is required^8,15^. This study aims to provide deeper insights into the research and healthcare priorities of Black, Asian, and other ethnic minority women, and those from the most deprived communities, by using a qualitative ethnographic, community-based approach to explore their experiences of accessing maternal healthcare, with the intention of guiding future research and action.

## METHODS

### Study design and setting

The current research was conducted using a qualitative ethnographic approach, with focus groups conducted in English and a focused-on participants’ cultural and familial needs. Semi-structured focus groups were conducted in person (4), in accessible community settings, e.g. libraries and mosques within Sheffield, United Kingdom, with participants children present where needed for childcare, and online on Zoom (3). Recruitment took place from April to August 2024. Relationships existed with communities through prior collaboration and were sustained through ongoing communication, shared goals, and mutual trust, which facilitated recruitment and engagement in the current study^16^.

### Research team and reflexivity

Interviews were conducted by A. Furness and H. Soltani. Both had a prior working relationship with a small number of participants who supported recruitment. All participants were informed of the research aims and interviewer’s professional background. Reflexivity was supported through team discussions and member checking.

### Participants

The study sample included 55 women recruited from areas of high deprivation with varied migration and ethnic backgrounds via purposive sampling techniques. The study was advertised via posters, professional organizations, local networks, Facebook, and community groups. Participants were excluded if they were not Black, Asian, or of ethnic minority backgrounds, or born outside the UK. The inclusion criteria were intentionally broad to capture a diverse range of experiences. Women not fluent in English were encouraged to participate, with other participants and community or group leaders assisting as translators. Participants received an information sheet and a consent form which was signed electronically or in person. A £10 gift voucher was given for participation, and confidentiality was maintained through pseudonyms.

### Data collection

Data were collected via the use of semi-structured focus groups. An interview guide was created and piloted by the authors that included prompts. Focus groups were audio-recorded and transcribed verbatim. Recruitment continued until data saturation was reached and no new topics emerged after the seventh focus group. Mothers were asked about their positive and negative experiences of accessing care in the UK for their children and what they wanted or felt could be improved.

### Data analysis

Qualitative data were analyzed using NVivo software and inductive thematic analysis. Triangulation was achieved by having two researchers independently coding the transcripts, with discussions to ensure credibility. The team then identified patterns, constructs, and themes, validating findings through participatory workshops with participants. The themes were derived inductively from the data and direct quotations were used to support interpretations.

## RESULTS

### Study participant characteristics

**Figure 1:**
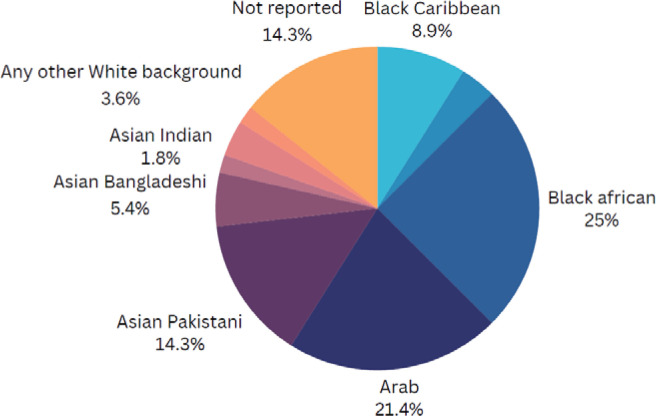
Ethnicity of participants in the UK

**Figure 2:**
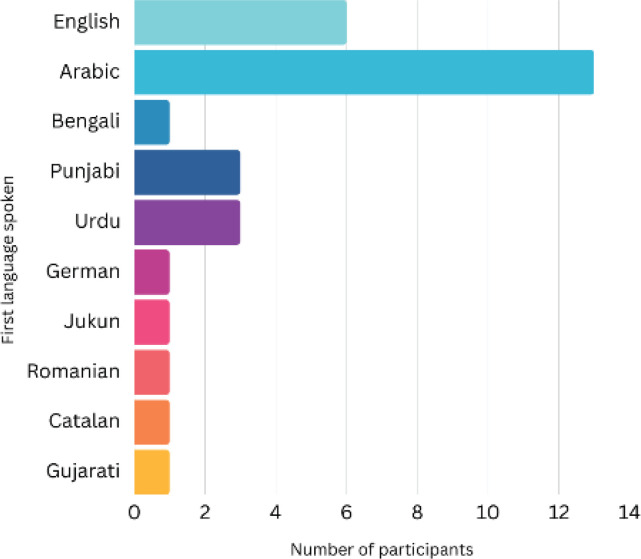
First language spoken by participants in the UK

**Figure 3:**
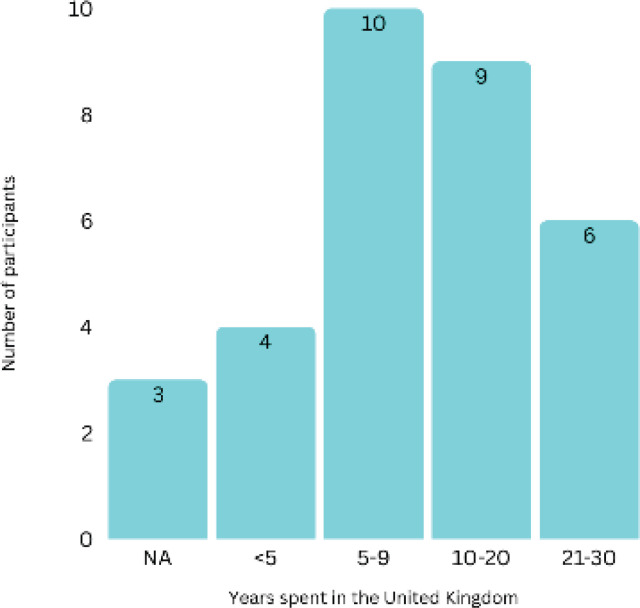
Number of years participants have spent in the UK

The study sample included 55 women from diverse ethnic backgrounds, with the largest groups identifying as Black African, Arab and Pakistani (Figure 1). Participants spanned various age groups (range: 20–49 years) (Supplementary file Figure 1). Thirty-five women were born outside the UK, and 15 participants had lived in the UK for 10 or more years (Figure 2). English was the first language for only 9 participants, with Arabic being the most common non-English language (Figure 3). Most participants had multiple children, with 19 having three children and only one currently pregnant (Supplementary file Figure 2).

### Themes and subthemes

**Figure 4:**
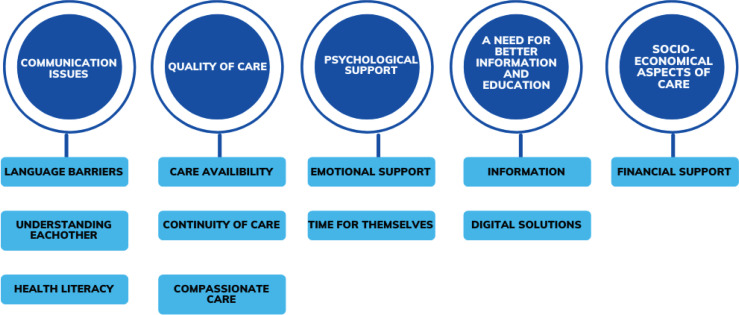
Themes and subthemes identified

Five themes were generated from the data as illustrated in Figure 4 with their related subthemes. Each is discussed in turn below.

*Communication issues*

During the research, women reported that communication barriers hindered timely access to and effective use of healthcare, especially for those in greatest need. These barriers extended beyond language difficulties to include limited health literacy and a lack of culturally competent communication.

Language barriers

A recurrent subtheme among participants was language difficulties when communicating with HCPs. Many relied on family members as translators, which often meant scheduling appointments around their availability, particularly work commitments. Others expressed concern for people accessing healthcare who did not speak the language, due to their negative experiences:

*‘I’ve got same problem I’ve faced in my pregnancy about the language. I speak Urdu … when I was pregnant always my husband attended a GP [general practitioner] and the midwives every time he will be with me.’*

*‘I literally had to come down to tears for someone to come up to me and they were like “the doctors were out, the file got missed”. I was asking for help, and I feel sorry for people who have a language barrier that can’t call out … I’m somebody that doesn’t have that barrier and I’m still treated like that.’*

Additionally, one mother highlighted the difficulty of learning English to improve communication with healthcare providers (HCPs), explaining that attending language lessons was challenging while caring for her children.

Understanding each other

Others describe difficulties when using interpreters such as the interpreter misunderstanding complex issues which then would lead to a lack of understanding of HCPs:

*‘Sometimes it causes you know misunderstanding from both sides and then she just record whatever she feels what’s right and it’s wrong and what she recording is wrong.’*

Health literacy

Concerns regarding understanding of how the UK health system worked were widespread, despite some having lived in the UK for prolonged periods. There was a need for education and guidance on navigating healthcare and understanding services:

*‘I’m pregnant I’m still with my first because I didn’t know what to expect I’ve been living in England for three years, So I don’t have much experience.’*

*‘Because when I came to this country, I came pregnant. And the first time for the woman to be pregnant, you don’t know anything you know about pregnancy. Uh, I missed so many things in my pregnancy with my daughter.’*

One mother who ran a mother’s group suggested that women felt grateful for the care received in the UK due to it being described as superior in comparison to the country they were born:

*‘One thing we tend to find is women feel they need to be grateful for the services … How can I complain when we’re getting it for free? … and the care is exceptional compared [to their home country].’*

Women required further information about their childcare entitlements and ‘*that you’re seeing all the services, that you’re supposed to access*’.

*Quality of care*

Care availability

Some women described an absence of healthcare services, with several mothers revealing that HCPs had missed home visits or that appointments had been delayed. Other concerns arose around a reduction in healthcare appointments between pregnancies and the acknowledgement of the NHS systemic change, for instance reduced continuity of care, changes in pathways and NHS resource constraints.

Women reported fragmented care and difficulties accessing consistent healthcare services. One participant’s pneumonia was diagnosed late, with the sentiment that care is only provided ‘*when you are about to die’.*

Others indicated they struggled to manage their healthcare alongside caring for multiple children, often resulting in their appointments being missed or put off altogether:

*‘I had to go into the hospital and when you’re breastfeeding your own baby you don’t want to. Especially when you’ve already had children, you’ve got childcare to juggle.’*

Continuity of care

In addition to difficulties accessing services, mothers also experienced challenges in receiving consistent care due to frequent changes in providers. Many mothers felt continuity of care was lacking, leading to missed services and fragmented information:

*‘I found out after having my second child that my first my daughter. She’s missed out on her vitamin and no, the TB, the BSG vaccination.’*

*‘We were high risk this is where the information gets missed. Yeah, because I saw a different midwife each time I was. I was getting different pockets of information.’*

Mothers wanted to be able to build a relationship with their midwives and felt unable to due to the lack of continuity:

*‘My midwife was always changing, even within in the GP, whenever I needed to speak. It’s always somebody else. They weren’t always consistent. They were always different midwives. So it never felt personal.’*

*‘In Germany, you really have one midwife in the entire pregnancy, and they do care.’*

Women who had experienced the same midwife during all their pregnancies reported how ‘nice’ it was due to the midwife knowing their personal circumstances.

Compassionate, culturally sensitive and personalized care

Women desired a compassionate approach from their HCPs. Mothers described instances of negative experiences from HCPs during labor and pregnancy:

*‘There was no sympathy, no empathy, no understanding, they’re not really listening.’*

Others suggested HCPs were ‘pushy’ or ‘rude’ with mothers and had a lack of empathy. Some mothers suggested more training was needed for HCPs in their bedside manner and improved communication style:

*‘I reckon some training in bedside manner would be great.’*

*‘I think it’s just teaching them how to be nice to patients.’*

Negative experiences led to a lack of trust in HCPs, which in turn hindered the access to healthcare for women. Women also described feeling as if they were treated differently from their White counterparts:

*‘… because of my previous experience, not just with the midwives, but with the GPs as well. So I don’t trust them much.’*

*‘… at some point we face some mistrust or of the healthcare system. Sometimes it could be based on our you know our backgrounds … there is a little bit of stigmatization.’*

One mother described being met with hostility after asking for a female HCP to conduct her scan for religious reasons:

*‘I asked a female to do my scan and then the lady came up. She shouted. Really, you know, she said “we don’t have a female today you have to go back” and I had diabetes … I said to her, “look, it’s not about you, it’s about my religion”.’*

Women reported feeling left alone and neglected and these negative experiences affected their mental health. There were suggestions that midwives’ unempathetic attitude was due to long shifts, shift patterns and tiredness:

*‘And when you’ve seen a member of staff for 12 hours, just be moody, be not helpful. No, we understand that those periods of time, you know, she might be finishing now, and she’s just tired. We understand that.’*

*‘That’s why they call it vocational professions. If you’re not vocational, don’t do it.’*

*Psychological support*

Emotional support and a safe space to talk

Women expressed the importance of having a safe space to talk, they relied heavily on support from their own families. A few mothers reported seeking other external support from general practitioners, charities and community organizations:

*‘They used to provide me somebody to talk to me. One to one that helped me so much.’*

There was a desire to receive emotional support during pregnancy, particularly in the early stages, from an external individual. Peer support workers were suggested to help the women through their transition to parenthood. Furthermore, women placed a high value on social support during the perinatal period and suggested there was a requirement for more women’s groups to share stories and experiences of pregnancy and child development. These groups were especially important to act as a support network for women who may not have a family in this country. Safe spaces were wanted to be available in the local area within their community to make them easily accessible:

*‘Like doing a group with a professional. Advise them what to do in the in the first, in the first solution for pregnant mums.’*

*‘Maybe the second time around … you’re going through a lot of emotional change … you need help and support at that point.’*

Some participants who had experiences of hyperemesis gravidarum described needing more support than they received. During this period, they felt they could not rely on their families or usual HCPs, due to their mental health:

*‘Mine was so bad my first three months where our thinking. I want this baby gone. Because I suffered very bad sickness, sickness and nauseousness. But I would think if something happened to me, the baby, I was so traumatized by it.’*

*‘I think that the other three kids that I’ve had, the sickness. I always say if it wasn’t for my belief I would have been suicidal. Those thoughts were really evil … and because of that, it made me even shut out people even more.’*

This extended to women who suffered from postnatal depression. One mother described her struggle with postpartum depression after her child was discharged from the NICU:

*‘With the first one, I didn’t want her either. My depression was around her. I didn’t like her. I didn’t want her. I wasn’t bothered with her, nothing.’*

Time for themselves

The demands of childcare often lead parents to struggle to allocate time for themselves, especially when caring for multiple children with differing schedules and needs. Women wanted to meet other women in similar life stages for peer support. This sentiment was eloquently captured by one participant, who expressed the difficulty of balancing their responsibilities with personal priorities:

*‘It’s because, you know, people have multiple children, where they’re not all like me, where, you know, one is in full-time education … So it’s like, how can I juggle this? For it to fit so I can attend this thing, because I really need it for my mental health. But it’s just a bit of me time.’*

Exercise and being outdoors were seen as making time for themselves and as a form of self-care:

*‘[Exercise is] something for me, something that’s mine, something I can really take ownership of.’*

*‘I prefer to be outside in the nature, another place from stress.’*

*Need for better information and education*

Information for before, during and after pregnancy

Many participants expressed that they were not provided with sufficient information before, during, or after their pregnancies. First-time mothers particularly expressed a need for more pregnancy and labor education to reduce anxiety and help them prepare:

*‘But that’s just because I educated myself. I was still not given this information about anything.’*

Many women described feeling more confident in their second pregnancies as they knew what to expect:

*‘During my first pregnancy, I had no idea what to expect and needed help or advice.’*

Participants suggested that accessible and comprehensive education on pregnancy could help mothers feel more prepared and confident:

*‘I think research will help create awareness and cover knowledge gap on what women need to know especially what the need during pregnancy.’*

Transition to parenthood

Mothers also identified a lack of information beyond pregnancy, particularly in the early years of their child’s life:

*‘I think the time between one years until two years or three years old, we miss lots of information.’*

Reliable digital solutions

During the perinatal period, women turned to online sources (Google, social media) and sought guidance from friends and family. However, concerns were expressed over unreliable information sources and misinformation from social media. They expressed a preference for receiving reliable, evidence-based information directly from HCPs:

*‘Online should be direct from qualified healthcare personnel because anyone can just come in with the information and put online.’*

Some participants voiced concerns about the shift towards online maternity information, emphasizing the importance of in-person interactions:

*‘This move towards online education. I think it can’t be a replacement because that is harmful and one thing I find even with myself is I need that social interaction with my health professionals, other women and even antenatal education.’*

Participants desired more nutritional information, particularly about healthy eating during and after pregnancy, nutrient deficiencies, weight management and provision of healthy children’s meals. Culturally sensitive nutritional information was also desired, religious-specific nutritional information relating to fasting:

*‘There was a lot of things that I neglected myself and I wasn’t taking my medication properly, especially when I’ve been anemic quite a lot or, you know, vitamin D deficiency and like I was just I’m just delaying my medicine, I suppose, and now I’ve I’m just stuck with being anemic.’*

Women also stated they wanted information on physical activity during and after pregnancy including opportunities for walking and socializing:

*‘What I found with the GP is and when it comes to the actual midwives, there’s not much connection with community organizations that can offer the physical education. I don’t think the NHS has the capability to offer physical education, but I think they can work with community organizations.’*

*Socio-economical aspects of care*

Financial support for pregnancy needs

Participants highlighted the need for financial assistance to cover essential pregnancy-related expenses, which can burden many families. One participant mentioned the importance of such support for necessities like vitamins, emphasizing how these costs can add up and create additional stress during an already demanding time:

*‘I think I had issues. UM, financial issues. It wasn’t really easy, you know.’*

*‘I would like to talk about in terms of services because we have been going for in terms of services as pregnant women … it requires me to have a little kind of funds. In order for me to get certain nutrients like protein, so I really need support like in terms of to buy [food] … to make smart food choices that can help me have a healthy pregnancy and a healthy baby.’*

## DISCUSSION

People previously and wrongly identified as ‘hard to reach’ have been excluded from research, despite representing some of the most disadvantaged populations for maternal morbidity and mortality^1,2,13^. There has been a call for more representative research that includes seldom-heard communities to improve public health outcomes. Prioritizing their needs in research is crucial for developing targeted interventions that reduce maternal health and systemic disparities^17^.

The most notable finding of the present study was that women needed more emotional support during the perinatal period. Although this is likely to be the same for all women, perinatal mental health outcomes are predominantly worse amongst migrant women^18^. Despite this, Black African, Asian and ‘White Other’ women have lower access to community mental health services^19^. The literature highlights the need to evaluate women’s social support during pregnancy, with interventions potentially protecting against perinatal depression^11,20^. Women stressed the importance of having an HCP in a supportive capacity. However, barriers such as socio-economic, distrust of professionals and language difficulties mean this is not always possible^21^. Trained peer supporters for perinatal mental health have been suggested to bridge the gap between services and increase women’s feelings of friendship^20,22^. There are instances where women experience higher levels of isolation such as when seeking asylum^23^. During the transition to motherhood, all women need strategies to build a support network, poor levels of support may increase the risk of adverse outcomes for both mother and child^20^.

Consistent with existing literature, this research identified language barriers and communication challenges with HCPs as obstacles for women^6-8^. Effective communication is crucial for safe healthcare, while ineffective communication hinders access to culturally safe and inclusive care^6^. This has been demonstrated in early infancy where serious interpretation failures have been identified in at least 80 instances of infant death and injury between 2018–2022^24^. Within our study, women primarily relied upon their family members or friends as translators. While this approach can be effective, the NHS advises caution due to the lack of professional training, which may impact the accuracy of the consultation^25^. Furthermore, the General Medical Council stipulates that a professional interpreter should always be offered to ensure equitable access to maternity services and mitigate disparities in maternal health outcomes^26^.

There was a notable acknowledgement of the absence of perinatal information for ethnic minority women. This led women to non-professional sources of information, such as social media, the internet and peers^27^. Immigrant women have been shown to use social media and WhatsApp groups to find information to prepare for birth and the postpartum period^27^. Social networking sites have been shown to have a direct impact on women’s decision-making during pregnancy^28^. Reputable information must be present on social networking sites, perhaps provided by HCPs. Research indicates positive feedback with supported interventions delivered over social media platforms^29^. Our research underpins the necessity for accessible information to be provided online in multiple languages, where HCP-provided information is absent.

Participants highlighted an absence of antenatal care services within their local community, a gap that may reflect broader systemic issues in service provision. The WHO asserts that all women should have access to antenatal care and have a minimum of eight sessions^30^. Previous research shows that women from ethnic minority backgrounds accessed antenatal education late, leading to inequities, and highlighting the need to improve access to antenatal classes in areas with high ethnic diversity and social deprivation^31^.

Mothers highlighted receiving a paucity of nutrition and physical activity information. The antenatal period is associated with a decline in physical activity^32^. Midwives are the primary source of information for women during pregnancy; however, research stipulates they feel undertrained in guiding physical activity, leading women to such online resources^32^. Studies have reported increased confidence and motivation of HCPs to engage in discussions of physical activity after providing physical activity training^33^. Similarly, midwives feel they are responsible for nutrition education for women during the perinatal period^34,35^. Similar to our research, the literature shows that information gathered online for prenatal nutrition is often inaccurate and hard to read^36^. Women turned to apps and social media to aid their nutrition decisions^34^. Currently, research lacks an understanding of the information ethnic minority women receive during the perinatal period. Our findings underscore the necessity for tailored nutritional education amongst mothers.

Women wanted improved access to healthcare. Health literacy encompasses the knowledge, skills, and motivation to access and use health information effectively. Immigrants often struggle to navigate the UK healthcare system, increasing their risk of poorer obstetric outcomes with access further complicated for undocumented women^2,23^. Several reports have shown a scarcity of interventions to improve health literacy in the UK, particularly for those with limited English proficiency^9^. Improving health literacy and trust in healthcare among ethnic minority women is key to enhancing access and promoting health equity.

### Strengths and limitations

The primary strength of this study lies in its authentic community engagement and participatory approach, involving a relatively large and diverse group of participants. Although the sample was diverse, the experiences of the participants may echo the priorities and needs of ethnic minorities but also all women living in areas of deprivation. Future studies should try to understand what may be truly unique for women who are in the minority. Although efforts were made to reach the most marginalized, the study primarily engaged those already connected to community networks, therefore it may not fully capture the perspectives of the most isolated or marginalized individuals.

## CONCLUSIONS

To address health inequalities, ethnic minority women identified key priority areas, including reducing communication barriers, improving the psychosocial aspects of care, and enhancing the quality and continuity of care to ensure cultural safety and sensitivity. There was an emphasis on the need for better information, particularly during the transition to parenthood. Additionally, they advocated for the use of advanced technology and digital platforms to expand access and improve healthcare experiences on a broader scale.

## Supplementary Material



## Data Availability

The data supporting this research can be found in the Supplementary file.
