# Supplementary material for: Exploring research and healthcare priorities in maternal health: A qualitative ethnographic study with mothers from ethnic minority backgrounds in the UK
Source: Eur J Midwifery. 2025 Sep 12;9:10.18332/ejm/209195. doi: 10.18332/ejm/209195 (PMC12426954; doi:10.18332/ejm/209195)
Supplement: Supplementary file 1 [file EJM-9-42-s1.pdf]

Supplementary file

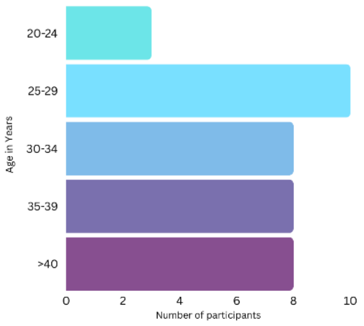

Figure 1: Age of participants UK.

Study design: Qualitative ethnographic study; Sample size: n=55; Year: 2024; Setting: Community-based focus groups with mothers from ethnic minority backgrounds in South Yorkshire.

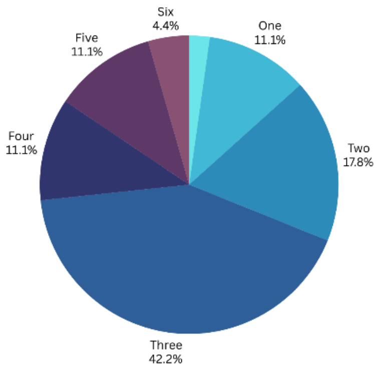

Figure 2: Parity UK.

Study design: Qualitative ethnographic study; Sample size: n=55; Year: 2024; Setting: Community-based focus groups with mothers from ethnic minority backgrounds in South Yorkshire.

© 2025 Furness A.M. et al.
